# Injury of the Tibial Nutrient Artery Canal during External Fixation for Lower Extremity Fractures: A Computed Tomography Study

**DOI:** 10.3390/jcm9072235

**Published:** 2020-07-14

**Authors:** Haidara Almansour, Johann Jacoby, Heiko Baumgartner, Marie K. Reumann, Konstantin Nikolaou, Fabian Springer

**Affiliations:** 1 Department of Diagnostic and Interventional Radiology, Tuebingen University Hospital, Eberhard Karls University Tuebingen, Hoppe-Seyler-Str. 3, 72076 Tuebingen, Germany; haidara.al-mansour@med.uni-tuebingen.de (H.A.); konstantin.nikolaou@med.uni-tuebingen.de (K.N.); 2 Institute of Clinical Epidemiology and Applied Biometry, Eberhard-Karls-Univerisity Tübingen, Sicherstr. 5, 72076 Tübingen, Germany; Johann.Jacoby@med.uni-tuebingen.de; 3 Department of Trauma and Reconstructive Surgery, BG Unfallklinik Tuebingen, Eberhard Karls University Tuebingen, Schnarrenbergstr. 95, 72076 Tuebingen, Germany; hbaumgartner@bgu-tuebingen.de (H.B.); mreumann@bgu-tuebingen.de (M.K.R.); 4 Department of Diagnostic Radiology, BG Unfallklinik Tuebingen, Eberhard Karls University Tuebingen, Schnarrenbergstr. 95, 72076 Tuebingen, Germany

**Keywords:** external fixation, nutrient foramen, tibia, computed tomography, tibial fracture, safe corridors, computed tomography, iatrogenic

## Abstract

The tibial nutrient artery (TNA) is the major diaphyseal artery of the tibia supplying two thirds of the inner osseous cortex. Hence, iatrogenic injury of the TNA endangers the integrity of the tibial blood supply and may compromise fracture healing. The incidence of its injury in the setting of external fixation for lower limb fractures has not been previously investigated. The aim of this study was to evaluate the incidence of TNA injury in the context of external fixation and to characterize the topography of the fixator pins in relation to the TNA canal (TNAC). Patients who underwent external fixation for distal femoral fractures and for tibial (proximal, shaft, and distal) fractures and had a postoperative computed tomography study were retrospectively included. The following parameters were retrieved: 1) Pin characteristics (orientation and cortical position of the pins), 2) The anatomic relationship between the TNAC and external fixation pin (topography above/below and at the level of the TNAC, and the distance between the pin and medial tibial plateau and/or the medial malleolus), and 3) The incidence of TNAC injury (complete/partial disruption of TNA lumen). A total of 105 patients with 214 tibial pins were analyzed. In 27 patients (26%), the TNAC was completely injured by the pins of the external fixator. In 13 patients (12%), the TNAC was partially injured. Of the 214 analyzed pins, 85 pins (40%) were located at the level of the TNAC (the TNAC and the pin are seen on the same axial slice). Most pins that were applied at the level of the TNAC belonged to a knee-bridging external fixator. Of those, ninety-three percent of the pins were anteromedially applied according to published surgical guidelines. Six percent of the pins were applied through the tibial crest and 1% anterolaterally. Of those 85 pins, 42 pins (49%) injured the TNAC at least partially. Based on the analyzed pins and the incidence of partial and complete injury of the TNAC, we observed that the tibial segment at which the tibial nutrient artery is endangered was located approximately (95% CI: 13–15 cm) from the medial tibia plateau and (95% CI: 22–25 cm) from the medial malleolus. Thus, TNAC injury by external fixation pins in the context of lower limb fractures can be considered common. Almost half of the pins applied at the middle third of the tibia injured the TNA, despite adherence to published surgical guidelines for external fixation. When possible, pin application at the middle third of tibia should be avoided to circumvent iatrogenic injury of the TNA and to safeguard tibial blood supply.

## 1. Introduction

The tibial nutrient artery (TNA) is the main artery of the tibial diaphysis supplying the bone marrow and the inner two-thirds of the cortex [1,2,3,4]. In most cases, a single TNA exists, and only very rarely can two or three nutrient artery canals be observed along the tibial diaphysis [4,5,6]. The tibial diaphysis has a poor extraosseous blood supply at its posterior aspect, leaving the TNA to be the key player for providing adequate blood supply, especially in the context of fracture healing when increased blood supply is needed most [7]. Injury to the nutrient arteries of long bones has thus been reported as a risk factor for delayed or non-union due to the depression of callus formation [2,3,4,7,8,9,10]. In addition, the integrity of the TNA is very important during childhood, and its injury could lead to growth impairment due to a disruption of blood supply to the epiphyseal plate, as it provides more than 70% of intraosseous blood supply [4,11]. As a result of this artery’s significance, the preservation of the TNA during surgical procedures has been previously advocated [3,9,10].

The TNA may be iatrogenically injured via internal/external fixation devices [1,4,12]. In the context of acute trauma, external fixation for lower limb fractures is an essential tool in the armamentarium of the trauma surgeon [13,14]. The main indication is “damage-control” via temporary fracture stabilization. The goal is to safeguard and reconstruct alignment, length, and rotation of the fractured limb [13]. However, vascular injury is considered one of external fixation’s complications, whose incidence is not well understood [13,15,16]. Despite the importance of the TNA, we are aware of only one cadaveric study that quantifies the incidence of the TNA injury in the context of intramedullary nailing in twelve human tibiae [12]. To the best of our knowledge, the incidence of the iatrogenic TNA injury in the setting of external fixation for lower limb fractures has not been previously investigated.

The aim of this study was to evaluate the incidence of TNA injury and to characterize the topography of the fixator pins in relation to the TNA canal.

## 2. Materials and Methods

### 2.1. Study Population

**Figure 1:**
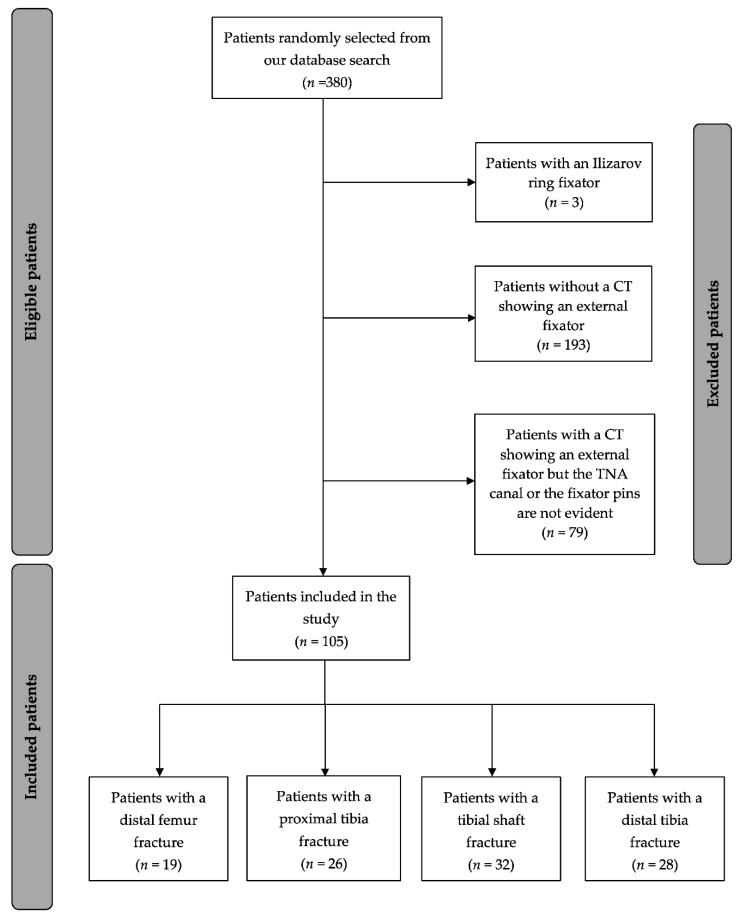
Flow diagram of the inclusion/exclusion process.

This retrospective study was approved by the institutional review board, which waved the requirement for informed consent. A database search revealed 2841 patients for which removal of an external fixation device was coded as a surgical procedure between 2013 and 2018. Patients with the following ICD-codes were retrieved (Distal femur fracture: 5–787.9 h, proximal tibia fracture: 5–787.9 k, tibial shaft fracture: 5–787.9 m, and distal tibia fracture: 5–787.9 n). In order to be able to include patients who underwent surgery before they were transferred to our hospital for definitive treatment. In the next step, patients were included in the presented retrospective analysis if they had a postoperative computed tomography (CT) study electronically available on the PACS (Picture Archiving and Communication System). CT examinations that did not illustrate at least one of the TNA nutrient foramina (allowing for assessment of the proximal or distal end of the TNAC) and at least one pin of the applied external fixator were excluded, in order to reliably assess the integrity of the TNA. Figure 1 illustrates the inclusion/exclusion process of our patient population. The first author (H.A.) was not involved in the clinical care of the included patients.

### 2.2. Imaging Protocol

CT acquisition was performed on a 128-slice high-end single source CT scanner (Somatom Definition Edge, Siemens Healthineers, Erlangen, Germany). Acquisition parameters were: 120 kV tube voltage, 90 mAs tube current, pitch 0.8, and rotation time 1.0 s, reconstructed slice thickness was 2 mm with standard filtered back projection, and no interative reconstruction algorithms were used.

### 2.3. Radiologic Assessment

**Figure 2:**
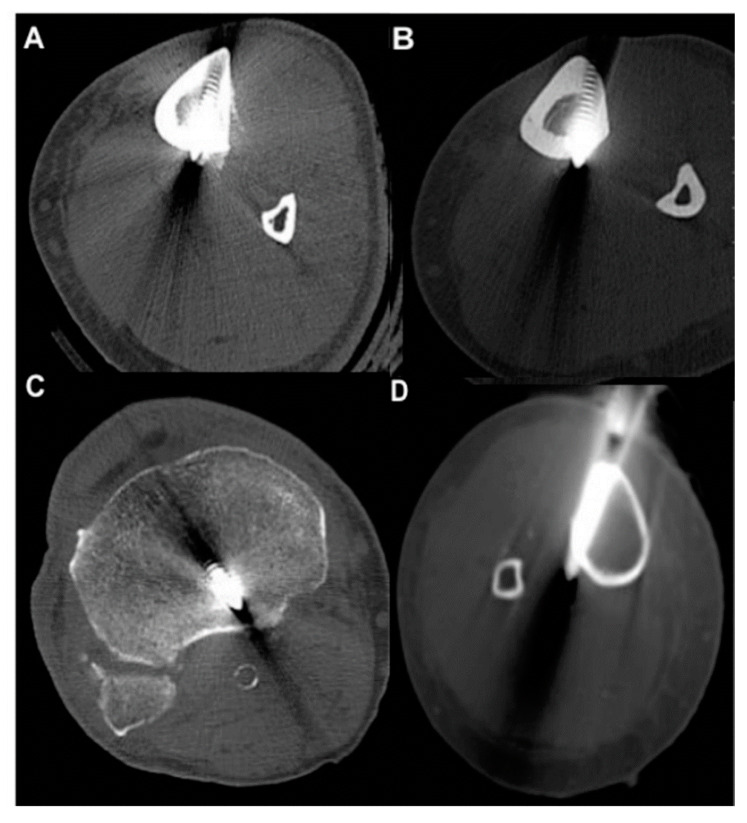
Axial reformatted images in bone window showing: (**A**) Anterior pin application through the tibial crest, (**B**) anterolateral pin application, (**C**) monocortical pin application, and (**D**) transcortical pin application.

**Figure 3:**
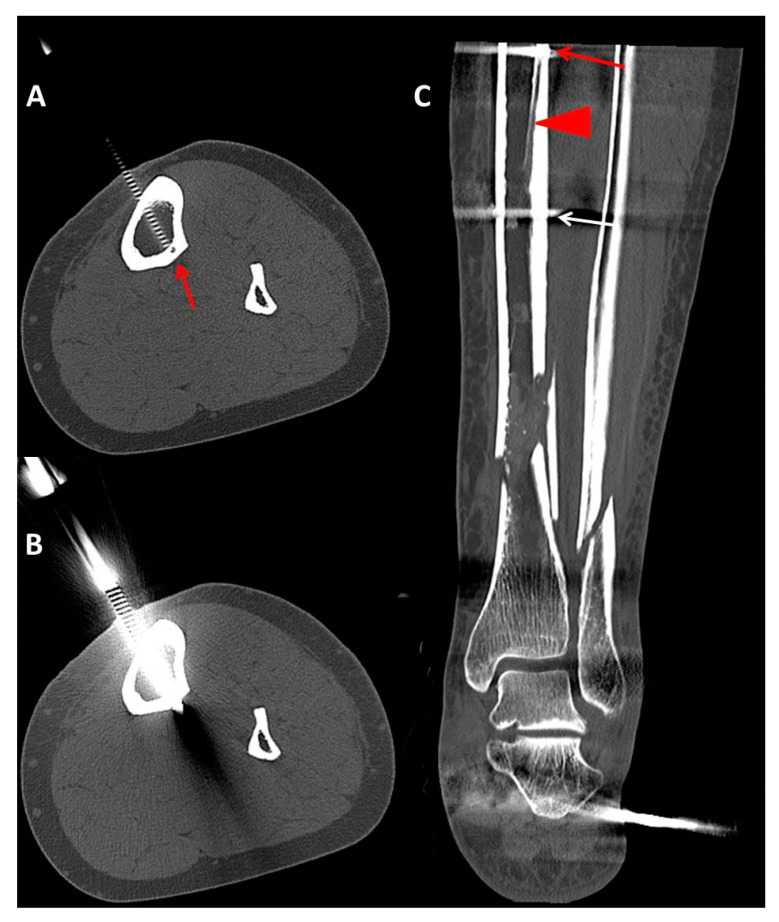
Subsequent axial (**A**,**B**) reformatted images in the bone window showing complete disruption of the tibial nutrient artery (TNA) canal (red arrow) by the proximal pin of an ankle spanning external fixator in the setting of a distal lower extremity fracture. (**C**) Coronal reformatted image illustrating both proximal (red arrow) and distal (white arrow) pins of the external fixator. The inner foramen of the TNAC is shown (tip of the red triangle). The outer foramen is not illustrated. Note that the proximal pin is applied in concordance with published surgical guidelines (i.e., bicortical and anteromedial application).

**Figure 4:**
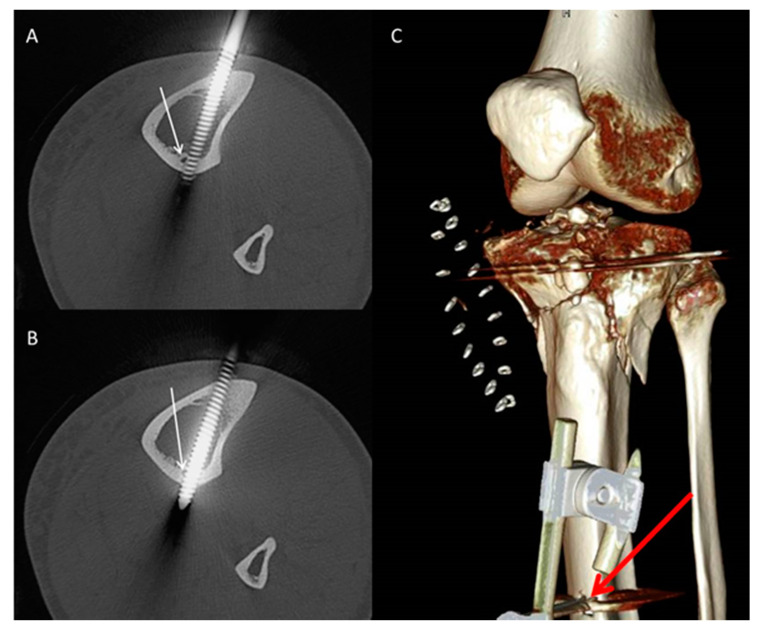
Subsequent axial (**A**,**B**) reformatted images in the bone window showing partial disruption of the tibial nutrient artery canal (white arrow) by the proximal pin of a knee spanning external fixator. (**C**) Three-dimensional reconstruction illustrating the anteromedial application of the pin (bold red arrow).

The following parameters were retrieved:

1. Pin characteristics (orientation of pin application: anteromedial/tibial crest, anterolateral) and cortical position of the pins (bicortical/monocortical/transcortical) (Figure 2).
2. The anatomic relationship between the TNAC and external fixation pin (topography above/below and at the level of the TNA canal, and distance between the pin and medial tibial plateau and/or the medial malleolus) for different fixation and fracture types.
3. The incidence of TNAC injury: (a) Complete injury: the TNAC lumen was completely disrupted by the pin (Figure 3); (b) partial injury: the TNAC lumen was tangentially disrupted (Figure 4).

### 2.4. Statistical Analysis

Descriptive statistics were calculated and reported as frequencies for categorical variables and as mean ± standard deviation (SD) for continuous variables. Chi-square and Fisher’s exact test were utilized to calculate statistical differences. The threshold for statistical significance was set at 0.05. SPSS v.26 (Armonk, NY, USA) was utilized for a statistical analysis.

## 3. Results

Following the above-mentioned inclusion process, a total of 105 patients (72 males, 33 females), aged 50 ± 16 years (range 17–85), were included in the current study.

### 3.1. Patient-Based Analysis

Most patients had a single TNA canal and only one patient had a double canal. In total, 53 knee-bridging external fixators, 34 ankle-bridging fixators, and 18 unimodular non-bridging fixators were studied.

In 27 patients (26%), the TNA was completely injured by the pins of the external fixator. In 13 patients (12%), the TNA was partially injured. Table 1 and Table 2 show the incidence of the TNA in all patients in terms of fracture type and external fixator type.

### 3.2. Pin-Based Analysis

#### 3.2.1. All Pins

In total, 214 tibial pins were analyzed. In most patients, two pins were depicted on the CT (60 patients, 57%), followed by one pin (26 patients, 25%), four pins (11 patients, 10.5%), and three pins (8 patients, 7.5%). Ninety-nine pins (46%) were applied proximal to the TNA canal and thirty pins were distal to the canal. Eighty-five pins (40%) were located at the level of the TNAC (the TNAC and the pin are seen on the same axial slice). Eighty-two percent of all pins were anteromedially applied, while 10% were applied through the tibial crest, and 8% were applied anterolaterally. Eighty-four percent of pins were bicortical, 13% were transcortical, and 3% of pins were monocortical (Figure 2). Of the 214 pins, 85 (40%) pins were located at the level of the TNA, and 129 (60%) were placed above or below the TNA (Table 3). Most pins that were applied at the level of the TNAC belonged to a knee-bridging external fixator. The fixator with the least number of pins applied at the level of the TNAC was a uni-modular non-bridging external fixator (Table 3).

#### 3.2.2. Pins Applied at the Level of the TNAC

Of the 85 pins at the level of the TNA canal, 79 pins (93%) of the pins were anteromedially applied according to published surgical guidelines. Five pins (6%) were applied through the tibial crest and one pin (1%) was applied anterolaterally. There was no statistically significant difference regarding the orientation of the pins in terms of TNA injury (*p* = 0.1). Seventy-three pins (86%) were bicortical, 10 pins (12%) were transcortical, and 2 pins (2%) were monocortical.

Of those 85 pins, 42 pins (49%) injured the TNAC at least partially. Table 4 and Table 5 show the distribution of pins that were applied at the level of the TNAC and the incidence of the TNAC injury according to the fracture type and external fixator type. The TNAC was mostly injured by fixator pins applied for proximal tibia fractures (64%), followed by tibial shaft (52%), distal tibia (46%), and distal femur fractures (31%).

### 3.3. Location of the Tibial Segment at Which the TNA Was Mostly Injured

Based on the analyzed pins and the incidence of partial and complete injury of the TNA, we observed that the tibial segment at which the tibial nutrient artery is endangered was located approximately (95% CI: 13–15 cm) from the medial tibia plateau and (95% CI: 22–25 cm) from the medial malleolus.

## 4. Discussion

Delineating the incidence of the iatrogenic TNAC injury has a crucial clinical relevance in the context of radiologic evaluation, preoperative planning, and fracture healing. This is the first study that quantifies the incidence of the iatrogenic TNA injury in the setting of external fixation for lower limb fractures.

### 4.1. The Significance of the TNAC and the Sequelae of Its Injury

To understand the significance of the TNA and the consequences of its injury, it is fundamental to understand the physiology of fracture healing and tibial angioarchitecture. Local vascular response to a fracture comprises five steps [6]. Firstly, blood flow is interrupted by direct injury. Secondly, vasoconstriction ensues and could lead to a 50% decrease in blood flow in the first 10 min and up to 30% of suppressed blood flow in the first 4 h as shown by an in vivo animal study analyzing canine osteotomies. Thirdly, enhanced blood flow occurs via vascular recruitment. Lastly, neoangiogenesis and remodeling can be observed. This process of revascularization could ensue via the extraosseous, intercortical, periosteal, and endosteal blood supply [6,17,18,19].

Tibial vascular supply consists of three systems: the epiphyseal-metaphyseal system, the periosteal system, and the nutrient artery. The epiphyseal-metaphyseal system comprises perforating arteries from the neighboring major arteries (anterior and posterior tibial arteries (ATA and PTA)) and its branches [5,7]. The outer third of the diaphyseal cortex is supplied by the periosteal system, which axially penetrates the cortex at fascial attachments and anastomoses with the nutrient arteries [2,5,6], whereas the inner two thirds are supplied by the TNA [1,3]. However, the tibial diaphysis is considered to be hypovascular due to its poor extraosseous blood supply, especially at its posterior aspect, rendering the nutrient artery to be the major diaphyseal artery [6,7]. The TNA usually originates from the anterior part of the posterior tibial artery and penetrates the bone through a groove on the posterior aspect of the tibia [2,7]. Most people have a single TNA, but multiple TNAs or the absence of a TNA have also been reported; however, this can be considered rare [4]. The outer nutrient foramen is located on the posterolateral aspect of the proximal third of the tibia. The inner nutrient foramen lies at the middle third of the tibia. The TNA canal mostly has a cranio-caudal oblique orientation [1,2,3,4]. In an analogous manner, tibial vascularity decreases in cranio-caudal fashion [20], with the upper third having the highest vascularity and the distal third having the lowest vascularity. This could be explained by the point-of-entry of the TNA at the upper third of the tibia as well as the arterial network of the knee supplied by the ATA and PTA. The distal third of the tibia is only vascularized via terminal branches of the TNA and some branches from the ATA [20]. This anatomical difference lends support to the contention that a fracture at the proximal third or an iatrogenic disruption of tibial blood supply in this region cuts off blood supply to the shaft where it is most needed for fracture healing, which, in turn, might be more predisposing to delayed or non-union than other tibial parts [20].

It has already been suggested that fractures in the distal third of tibia is more susceptible to increased rates of delayed or non-union due to the rupture of the TNA [21,22]. The region of the distal tibia has been acknowledged by many authors to be more susceptible to delayed fracture healing [23,24,25,26]. In an analysis of 416 surgically treated tibia shaft fractures, Audige et al. observed that for distal tibia shaft fractures, it was twice as likely to observe a delayed or non-union in comparison to other tibial shaft fractures. Similarly, in 245 surgically treated tibial shaft fractures with approximately six years follow-up, Bilat et al. observed 18 delayed unions in fractures traversing the middle or distal tibia shaft [24]. Indeed, the delayed healing or non-union is a multifactorial process and cannot be simply attributed to one factor like the injury of the TNA. Nevertheless, the topographic location should raise suspicion that injuring the major diaphyseal artery might play a role in this complex process [23]. However, the incidence of TNA injury was not discussed as a potential cofactor, and this phenomenon was attributed to postoperative fracture diastasis and the fact that the external fixator is less able to produce an optimal fracture alignment in comparison to open reduction and internal fixation (ORIF) [23].

### 4.2. External Fixation in the Setting of Lower Extremity Fractures and Its Perils

Lower extremity fractures have an alarmingly high incidence in up to almost 20% of patients with polytrauma [27,28]. These fractures constitute a major challenge as they are usually concomitant with multiple injuries and soft tissue damage.

Clinical decision-making is contingent upon soft-tissue injury, patient characteristics, fracture morphology, and topology [7,8,29,30]. There is a wide array of treatment options ranging from nonoperative treatment to external fixation, intramedullary nailing, and ORIF [7]. Intramedullary nailing is reserved for tibial diaphyseal fractures, while ORIF is usually performed for complicated intraarticular fractures [7,8]. However, provisional damage control requires the use of an external fixator, which allows for temporary fracture stabilization [27,31]. This method allows for the reduction of post-traumatic swelling of soft-tissues and the healing of superficial skin abrasions before definitive stabilization. It is considered a quick and efficient method, which can be used in dire and austere circumstances without the aid of intraoperative imaging [29]. In an international survey of 444 orthopedic surgeons associated with the Arbeitsgemeinschaft für Osteosynthesefragen (AO) foundation or the international members of the Orthopedic Trauma Association, Bhandari et al. [32] revealed that the use of external fixators was preferred more by surgeons in the context of high-energy trauma and fractures associated with compartment syndrome than in low-energy trauma. Furthermore, the more severe the soft-tissue injury was, the more surgeons were inclined to use an external fixator. Finally, surgeons without dedicated fellowship-training in trauma surgery were more likely to choose external fixation than intramedullary nailing, and vice versa. The relevance of this is that due to the lower technical aptitude required for external fixation; surgeons with less experience are more likely to choose external fixation as a primary fracture stabilization method. In contrast, surgeon’s experience is vital to achieve satisfactory postoperative results [13]. The external fixator was described in the literature as the “notorious non-union apparatus” [33]. Using a multi-planar external fixator as a definitive treatment, Gershuni et al. reported about 41% of non-union in 40 tibial fractures [34]. Clifford et al. reported about approximately 24% non-union in a sample of 42 patients [35]. Helland et al., using a uniplanar external fixator, reported a 14% non-union rate in a sample of 50 patients [36].

Vascular injury and non-union in the setting of lower leg fractures has been previously explored [37,38,39], encompassing arterial occlusion, pseudoaneurysms, and vessel wall damage [30,40]. Dickson et al. showed the interdependence of sound fracture healing and intact vascular supply in a retrospective analysis of 114 patients who underwent arteriography post tibial fractures caused by blunt trauma. The authors showed that patients with an injury to the anterior tibial, posterior tibial, or peroneal artery (52 patients) were significantly more likely to develop delayed or non-union [39]. Of those patients, 19 patients underwent external fixation as an index operative treatment. Similarly, in a retrospective analysis of 29 tibial shaft fracturs, Brinker et al. observed that injury to the posterior tibial artery is a significant risk factor for delayed and non-union. The authors argued that one of the reasons was disruption of the TNA being a branch of the posterior tibial artery and, consequently, the suppression of blood supply as a reason for the observed impaired fracture healing [37].

On the other hand, rates of iatrogenic vascular injury, especially during external fixation, are understudied [16] and underreported [15,40], and are sometimes discrepant and mostly published in the form of case reports [40]. For instance, the only study on TNA injury was conducted by Paar et al., which analyzed the rate of disruption of TNA in 12 human tibiae, and applied reamed (6 tibiae) and unreamed (6 tibiae) intramedullary nailing. The authors showed that the TNA was injured in all specimens that underwent reamed intramedullary nailing and was at least partially disrupted in 50% of the specimens that underwent unreamed intramedullary nailing. Paul et al. conducted one of the largest studies examining iatrogenic extraosseous vascular injury in the setting of external fixation and observed four cases of iatrogenic injury out of 121 tibial and femoral fractures (3.3%) [15,40]. Dhal et al. reported that the most common cause of pseudoaneurysm in their case series (38.5%) was the iatrogenic injury of the external fixator pins [16,41].

### 4.3. Safe Application of External Fixation Devices

The principles of a successful external fixation underlie anatomical and biomechanical considerations as well as patient-specific and fracture morphologic prerequisites [29]. The number, orientation, size, and distance of pins to fracture fragments are important for the stability and safety of the construct [13,29]. The rigidity of the construct is increased by increasing the number and the distance between applied pins. Optimally, one pin should be as close to the fracture as possible and the other pin as far as possible, all while considering that a future incision for definitive treatment is required and while respecting extraarticular application [13,29]. This could explain why, in our study, iatrogenic TNA injury was different between the three external fixator and the four fracture types investigated. The TNA was mostly injured by fixator pins applied for proximal tibia fractures (64%), followed by tibial shaft (52%), distal tibia (46%), and distal femur fractures (31%).

Safe pin placement necessitates meticulous knowledge of cross-sectional anatomy [42]. The current guidelines, such as the Arbeitsgemeinschaft für Osteosynthesefragen (AO) surgery reference, recommend a bicortical and anteromedial approach, either just medial to the crest or perpendicular to the anteromedial surface, to avoid injury of neurovascular structures [43]. In our study, 82% of all pins were anteromedially applied, while 10% were applied through the tibial crest, and 8% were applied anterolaterally. Eighty-four percent of pins were bicortical, 13% were transcortical, and 3% of pins were monocortical. Of the 214 pins, 85 (40%) pins were located at the level of the TNAC. Most of the pins located at the level of the TNAC were applied according to published guidelines. Ninety-three percent of those pins were anteromedially applied, and 86% were bicortical. Despite adherence to those standards, the incidence of the cumulative TNAC injury was 49% (33% complete injury and 16% partial injury) (Table 4). Since the location of the TNA is relatively consistent, avoiding its injury could be circumvented via preoperative planning. In a previous anatomic study conducted on a central European population, we showed that the TNA canal crosses approximately the middle third of the postero-lateral tibia at a mean distance between the medial tibia plateau and the outer and inner TNA foramen of (11–12 cm) and (14–15.5 cm), respectively. The outer and inner TNA foramina were located at approximately 31% and 42% of total tibial length [4]. The mean total tibia length was 36 cm and 37 cm for females and males, respectively [4]. In the current study, we found that based on the analyzed pins and the incidence of partial and complete injury of the TNA, we observed that the tibial segment at which the tibial nutrient artery is endangered was located approximately (95% CI: 13–15 cm) from the medial tibia plateau and (95% CI: 22–25 cm) from the medial malleolus. This implies that for a tibial length of 39 cm (the estimated average in our sample), the segment would have a length of approximately 3.8 cm. When possible, surgeons should avoid pin application at approximately the middle third of the tibia to avoid iatrogenic injury of the TNA.

### 4.4. Limitations

The small sample size as well as the retrospective, single-center design and the inherent selection bias jeopardize the external validity of our results. Missing clinical information is a known limitation of retrospective studies. For instance, due to radiation dose reduction protocols, some of the CTs only included the fractured segment and not the entire lower leg or entire tibia. As a result, not all pins were evident on CT, and not all CTs revealed both the outer and inner foramina of the TNA. Furthermore, we could not calculate the mean total length of the tibia in this population as not all CTs revealed both the medial tibia plateau and the medial malleolus to allow for a reproducible measurement. Nevertheless, the mean total length of the tibia in a central European population has been previously studied [4] and may be extrapolated to this population. In terms of the statistical analysis, ideally, an analysis of different patients, fixators, fractures, and the likelihood of individual pins to injure the TNA canal would employ a mixed model approach. In such a model, pins would be conceptualized as observations nested within patients (and thereby within fractures and fixators) and, thus, potential dependencies among pin data could be adequately accounted for. However, the present data do not allow for such an analysis due to—not only, but most importantly—the retrospective nature of the study design and the fact of unequal distributions of the number of pins and their locations. The potential for dependencies is, however, quite small; one patient in the data set had fixators applied at both legs and yielded two observations in the data set. All other patients yielded only one observation. Nine patients out of 105 had two pins applied at the level of the TNA, and the remaining patients had one or zero pins applied at that level. We, therefore, conducted separate analyses on the patient level and at the level of potentially injuring pins. Thereby, even though the data do not allow to account for explicit modeling of dependencies in a full-fledged mixed model approach, our analyses minimize the potential for those dependencies to unduly influence results.

Indeed, pin placement within the known safe zones is imposed most importantly by the fracture pattern itself and by the vicinity of the major extraosseous neurovascular structures to the pin application site. Henceforth, this study does not attempt to define a more accurate safe zone, as this would require a prospective study within which patients undergo a CT exam encompassing the lower leg from the knee to the ankle. However, surgeons and radiologists should nevertheless be aware of the risks of pin application in the vicinity of the TNA canal and the commonality of its injury at the middle third of the tibia. Therefore, pin application remains at the discretion of the surgeon on a case by case basis.

## 5. Conclusions

In this study, TNA injury by external fixation pins in the context of lower limb fractures can be considered common. Almost half of the pins applied at the middle third of the tibia within approximately 13–15 cm from the medial tibial plateau and 22–25 cm from the medial malleolus injured the TNA canal despite adherence to published surgical guidelines for external fixation. When possible, pin application at the middle third of tibia should be avoided to circumvent iatrogenic injury of the TNA and to safeguard tibial blood supply.

## Figures and Tables

**Table 1:** The incidence of the TNAC injury according to fracture type for all patients.

Fracture Type	Total No. of Patients	Complete Injury		Partial Injury		No Injury		*p* *
		No. of Patients	%	No. of Patients	%	No. of Patients	%	
Distal femur	19	4	14.8%	1	7.7%	14	21.5%	0.12
Proximal tibia	26	11	40.7%	3	23.1%	12	18.5%	
Tibial shaft	32	4	14.8%	7	53.8%	21	32.3%	
Distal tibia	28	8	29.6%	2	15.4%	18	27.7%	
Total	105	**27**	**25.7%**	**13**	**12.3%**	65	30.3%	

* Fisher’s Exact test.

**Table 2:** The incidence of the TNAC injury in all patients according to the external fixator type.

External Fixator Type	Total No. of Patients	Complete Injury		Partial Injury		No Injury		*p* *
		No. of Patients	%	No. of Patients	%	No. of Patients	%	
Unimodular non-bridging	18	1	3.7%	4	30.8%	13	20%	0.16
Knee bridging	53	17	63%	5	38.5%	31	47.7%	
Ankle bridging	34	9	33.3%	4	30.8%	21	32.3%	
Total	105	**27**	**25.7%**	**13**	**12.3%**	65	30.3%	

* Fisher’s Exact test.

**Table 3:** External fixator type and topography of the applied pins in relation to the TNAC.

External Fixator Type	Total No. of Pins	At the Level of the TNA		Below/Above the TNA		*p* *
		No. of Pins	%	No. of Pins	%	
Unimodular non-bridging	56	13	23.2%	43	76.8%	**0.01**
Knee-bridging	92	43	46.7%	49	53.3%	
Ankle bridging	66	29	43.9%	37	56.1%	
Total	214	85	39.7%	129	60.3%	

* Fisher’s Exact test, bold denotes statistical significance. TNA: Tibial nutrient artery.

**Table 4:** The incidence of the TNAC injury via pins applied at the level of the TNAC according to the external fixator type.

Fracture Type	Total No. of Pins	Complete Injury		Partial Injury		No Injury		*p* *
		No. of Pins	%	No. of Pins	%	No. of Pins	%	
Distal femur	16	4	25%	1	6%	11	69%	0.069
Proximal tibia	22	11	50%	3	14%	8	36%	
Tibial shaft	23	4	17%	8	35%	11	48%	
Distal tibia	24	9	38%	2	8%	13	54%	
Total	85	**28**	**33%**	**14**	**16%**	43	51%	

* Fisher’s Exact test.

**Table 5:** The incidence of the TNAC injury via pins applied at the level of the TNAC according to the external fixator type.

External Fixator Type	Total No. of Pins	Complete Injury		Partial Injury		No Injury		*p* *
		No. of Pins	%	No. of Pins	%	No. of Pins	%	
Unimodular non-bridging	13	2	15%	4	31%	7	54%	0.39
Knee-bridging	43	17	39%	5	12%	21	49%	
Ankle bridging	29	9	31%	5	17%	15	52%	
Total	85	**28**	**33%**	**14**	**16%**	43	51%	

* Fisher’s Exact test.
